# Small fiber neuropathy for assessment of disease severity in amyotrophic lateral sclerosis: corneal confocal microscopy findings

**DOI:** 10.1186/s13023-021-02157-w

**Published:** 2022-01-06

**Authors:** Jiayu Fu, Ji He, Yixuan Zhang, Ziyuan Liu, Haikun Wang, Jiameng Li, Lu Chen, Dongsheng Fan

**Affiliations:** 1 Department of Neurology, Peking University Third Hospital, 49 North Garden Road, Haidian District, Beijing, 100191 China; 2 Beijing Municipal Key Laboratory of Biomarker and Translational Research in Neurodegenerative Diseases, Beijing, China; 3 Department of Ophthalmology, Peking University Third Hospital, Beijing, China

**Keywords:** Amyotrophic lateral sclerosis, Corneal confocal microscopy, Small fiber neuropathy, Corneal nerves, Inferior whorl length

## Abstract

**Background:**

Amyotrophic lateral sclerosis (ALS) is a fatal neurodegenerative disorder with progressive motor system impairment, and recent evidence has identified the extra-motor involvement. Small fiber neuropathy reflecting by sensory and autonomic disturbances in ALS has been reported to accompany the motor damage. However, non-invasive assessment of this impairment and its application in disease evaluation of ALS is scarce. We aim to evaluate the use of corneal confocal microscopy (CCM) to non-invasively quantify the corneal small fiber neuropathy in ALS and explore its clinical value in assessing disease severity of ALS.

**Methods:**

Sixty-six patients with ALS and 64 healthy controls were included in this cross-sectional study. Participants underwent detailed clinical assessments and corneal imaging with in vivo CCM. Using ImageJ, the following parameters were quantified: corneal nerve length (IWL) and dendritic cell density (IWDC) in the inferior whorl region and corneal nerve fiber length (CNFL), nerve fiber density (CNFD), nerve branch density (CNBD), and dendritic cell density (CDC) in the peripheral region. Disease severity was evaluated using recognized scales.

**Results:**

Corneal nerve lengths (IWL and CNFL) were lower while dendritic cell densities (IWDC and CDC) were higher in patients with ALS than controls in peripheral and inferior whorl regions (*p* < 0.05). Additionally, corneal nerve complexity in the peripheral region was greater in patients than controls with higher CNBD (*p* = 0.040) and lower CNFD (*p* = 0.011). IWL was significantly associated with disease severity (*p* < 0.001) and progression (*p* = 0.002) in patients with ALS. Patients with bulbar involvement showed significantly lower IWL (*p* = 0.014) and higher IWDC (*p* = 0.043) than patients without bulbar involvement.

**Conclusions:**

CCM quantified significant corneal neuropathy in ALS, and alterations in the inferior whorl region were closely associated with disease severity. CCM could serve as a noninvasive, objective imaging tool to detect corneal small fiber neuropathy for clinical evaluation in ALS.

**Supplementary Information:**

The online version contains supplementary material available at 10.1186/s13023-021-02157-w.

## Introduction

Amyotrophic lateral sclerosis (ALS), a fatal neurodegenerative disease, is characterized by progressive paralysis, dysphagia, respiratory failure, and eventual death [1]. The disease is considered to selectively impair the motor system [2]. However, recent studies have identified ALS as a multisystem disease with nonmotor involvement, including metabolic imbalance, sleep disorder, and peripheral neuropathies with minor sensory and autonomic disturbances [3–5]. Although the damage is to a much lesser extent than motor impairment, studies have reported clinical and electrophysiological results of sensory and autonomic damage in approximately 10–30% of ALS patients [6, 7]. The validation of facial-onset sensory and motor neuronopathy (FOSMN) as a rare phenotype of ALS also provides supporting evidence for this small fiber neuropathy, as it starts from trigeminal sensory disturbance to bulbar and limb weaknesses [8, 9]. Additionally, recent pathological studies have confirmed the small fiber neurodegeneration process through skin biopsy in both patients with ALS and animal models of ALS [10, 11]. Given the evidence that peripheral neuropathies can arise early in ALS, these changes could provide biomarkers to monitor disease progression and aid in treatment assessment from a novel perspective.

Corneal confocal microscopy (CCM) is a rapid, objective technique to detect peripheral small fiber neurodegeneration and inflammation in corneal structures at the cellular level by quantifying corneal nerve and dendritic cell architecture [12]. Due to dynamic growth in the corneal nerve plexus, the corneal nerves enter the tissue radially and migrate centripetally, resulting in a clockwise whorled pattern located slightly inferior to the corneal apex [13]. Thus, there are two representative regions for CCM to observe based on the corneal nerve pattern: the parallel peripheral area and the unique inferior whorl area [14]. In addition, immune cells known as dendritic cells can also be detected by CCM, which accompany corneal nerves and reflect the inflammatory responses maintaining corneal homeostasis [15]. As a noninvasive technique to visualize cell changes in vivo, CCM has been utilized to identify corneal nerve impairments in a range of central neurodegenerative disorders and peripheral neuropathies, including Alzheimer’s disease, Parkinson’s disease, familial amyloid polyneuropathy, and multiple sclerosis [16–19]. Ferrari et al. initially reported the small fiber neuropathy of the peripheral corneal area in 8 patients with ALS through CCM [20]. However, evidence of corneal impairments in the inferior whorl area in ALS is scarce. Besides, an exploration of its value for disease severity assessments with larger sample size is needed.

We aimed to use CCM to evaluate the characteristics of corneal nerves and dendritic cells in both the peripheral and inferior whorl areas in patients with ALS. Additionally, we aimed to explore the association between CCM parameters and the clinical severity of ALS to further investigate their value as imaging biomarkers.

## Materials and methods

### Study subjects

This cross-sectional single-center study was conducted at the Department of Neurology, Peking University Third Hospital (PUTH). A total of 130 subjects (66 definite and probable ALS patients and 64 healthy controls) participated in the study. Patients were diagnosed and classified based on the revised El Escorial criteria for definite or probable ALS [21]. Healthy control subjects were recruited and matched to the ALS group in terms of age and sex. The exclusion criteria for patients included a family history of ALS, refusal to participate in the study and patients with cognitive disorders. In addition, none of the participants had known eye diseases or systemic diseases known to affect corneal nerve and dendritic cell status (e.g., diabetes or rheumatoid arthritis). Subjects taking medications known to affect corneal integrity or with a history of contact lens wear were also excluded. All study participants were examined by baseline slit lamp examination before inclusion to avoid clinically significant corneal pathology. The study was approved by the institutional ethics committee of PUTH and adhered to the tenets of the Declaration of Helsinki. All the subjects provided written informed consent prior to inclusion in the study.

### Neurological assessment of ALS

All patients underwent a thorough set of neurological examinations and detailed electrophysiological examinations as a standardized routine for patients with ALS at PUTH [22, 23]. All patients with ALS were examined by experienced neurologists for motor neuron dysfunction, with at least two neurologists for each patient. The demographic and clinical information of all patients was recorded, including age, sex, age of symptom onset, site of symptom onset, disease duration, and other relevant data, as reported previously [4, 23]. We used the Revised ALS Functional Rating Scale (ALSFRS-R) to evaluate disease severity [24], and the rate of its decline was recorded as ΔFS for disease progression ((48-ALSFRS-R)/disease duration from symptom onset to the assessment) [25].

Based on the presence of upper motor neuron (UMN) and lower motor neuron (LMN) signs in the bulbar region, the patients were subdivided into 2 groups: ALS patients with bulbar involvement and ALS patients without bulbar involvement [26]. LMN signs included weakness, atrophy, fasciculation of bulbar motor neuron-innervated muscle, and affected muscles detected by electromyography. UMN signs included the presence of clonic jaw jerk, exaggerated gag reflex, exaggerated snout reflex and pseudobulbar features [21].

### Corneal confocal microscopy

Laser scanning in vivo CCM of the central cornea was performed in all participants using a Heidelberg Retina Tomograph III with the Rostock Cornea Module (Heidelberg, Germany) as previously described [17]. Each image represents a coronal section of the cornea of 384 × 384 pixels over an area of 400 × 400 μm, with a separation of 1 μm between adjacent images and a lateral resolution of 1 μm/pixel. The corneal subbasal nerve plexus around the peripheral cornea was scanned, and eight images (four for each side) of high clarity were randomly selected as pictures from the peripheral area. A distinctive whorl-like pattern was identified, and the three clearest images were selected as pictures from the inferior whorl area. All images were obtained by a single masked observer. Only the right eye of each participant was included for the analysis, as previous studies have shown no difference between the right and left eyes in both healthy controls and patients with neuropathy [16, 17]. To evaluate the parameters of CCM, semiautomatic Java-based image processing software (ImageJ, National Institutes of Health, Bethesda, MD, USA) and a plug-in (NeuronJ, Biomedical Imaging Group, Lausanne, Switzerland) were applied for tracing, quantification, and analysis. In the peripheral area, (i) corneal nerve fiber length (CNFL) represented the total length of nerve fibers and branches per frame; (ii) corneal nerve fiber density (CNFD) represented the total number of main nerve fibers divided by the area of the frame; (iii) corneal nerve branch density (CNBD) represented the total number of nerve branches divided by the area of the frame; and corneal dendritic cell density (CDC) represented the total number of dendritic cells divided by the area of the frame. In the inferior whorl area, (i) inferior whorl length (IWL) represented the total length of nerve fibers and branches per frame; inferior whorl dendritic cell density (IWDC) represented the total number of dendritic cells divided by the area of the frame. Two masked observers independently evaluated all the images, and the results for each measurement were averaged for further analysis.

### Statistical analysis

The qualitative data were reported as the number of participants, and the chi-square test was used for comparisons between groups. The quantitative data were reported as the means (standard deviation) and were initially tested for a normal distribution. Normally distributed variables between groups were compared using Student’s t test, and correlation analyses were performed using Pearson’s correlation. Nonnormally distributed variables were compared using Mann–Whitney U tests, while correlation analyses were performed using Spearman’s correlations. Statistical analyses were performed using SPSS 20.0 software (SPSS, Chicago, USA). The results were considered statistically significant at *p* < 0.05.

## Results

### Demographic and clinical characteristics

The baseline characteristics of the participants are given in Table 1. The ALS group consisted of 41 males and 25 females (mean age: 51.41 ± 11.19 years), and the control group consisted of 38 males and 26 females (mean age: 49.22 ± 10.34 years). There were no differences in age or sex distribution between the ALS and control groups (*p* > 0.05). The mean (SD) disease duration of the patients was 20.38 (12.02) months. The mean (SD) ALSFRS-R score was 38.85 (6.39), with a mean (SD) ΔFS of 0.57 (0.45). When further analyzed in subgroups, no differences were observed between the patients with and without bulbar involvement in terms of age, sex distribution and disease characteristics (*p* > 0.05; Additional file 1: Supplementary Table 1). All enrolled participants completed the study. The CCM procedure was well tolerated with no adverse events in any participant.

**Table 1:** Baseline characteristics of study participants

	Control, N = 64	ALS, N = 66	*P* value
Age, years	49.22 (10.34)	51.41 (11.19)	0.249
Sex, no. male/female	38/26	41/25	0.749
Disease duration, m	NA	20.38 (12.02)	NA
ALSFRS-R	NA	38.85 (6.39)	NA
ΔFS	NA	0.57 (0.45)	NA

Data are presented as mean (SD) values

ALS, amyotrophic lateral sclerosis; ALSFRS-R, Revised ALS Functional Rating Scale; ΔFS, (48-ALSFRS-R)/disease duration from symptom onset to the assessment; NA, not applicable

### CCM comparison between controls and patients

**Fig. 1:**
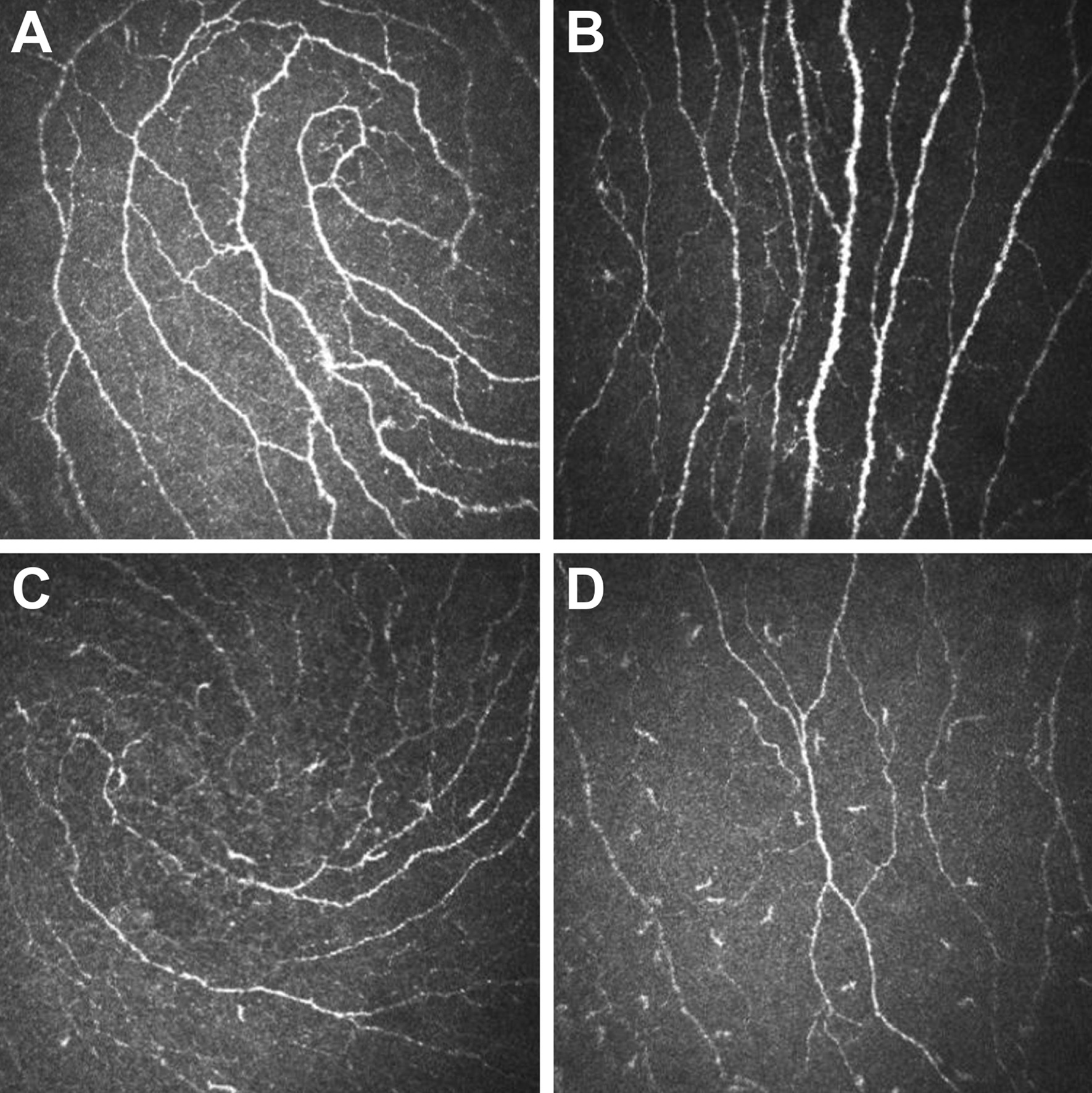
Representative CCM images of the inferior whorl area and peripheral area in control and ALS. In a control participant, the intact whorl-like pattern of the subbasal nerve plexus in the inferior whorl area (**a**) and the parallel nerve in the peripheral area (**b**) could be detected. In a patient with ALS, an impaired nerve pattern with increased dendritic cells in the inferior whorl area (**c**) and in the peripheral area (**d**) could be detected. CCM, corneal confocal microscopy; ALS, amyotrophic lateral sclerosis

As summarized in Table 2, there was a significant trend toward lower nerve fiber length and higher dendritic cell density in the ALS group than the control group in both peripheral and inferior whorl areas (*p* < 0.05). In the inferior whorl area, IWL was significantly lower, while IWDC was significantly higher in the patients with ALS than the controls (*p* = 0.014 and *p* = 0.020). In the peripheral area, the ALS group had a significantly lower CNFL (*p* = 0.017) with a significantly more complex nerve pattern, as the CNFD (*p* = 0.011) was lower while the CNBD (*p* = 0.040) was higher in the ALS group than in the control group. A significantly higher CDC was also observed in the peripheral area of patients than of controls (*p* = 0.019). Figure 1 illustrates representative CCM images of peripheral and inferior whorl regions in a patient with ALS and a healthy control subject.

**Table 2:** CCM parameters in controls and patients with ALS

		Control, N = 64	ALS, N = 66	*P* value
*Inferior whorl area*				
Corneal Nerve	IWL (mm/mm^2^)	19.96 (3.05)	18.33 (3.14)	0.014
Dendritic Cell	IWDC (/mm^2^)	20.02 (9.09)	36.83 (31.77)	0.020
*Peripheral area*				
Corneal Nerve	CNFL (mm/mm^2^)	19.52 (2.50)	17.80 (3.79)	0.017
	CNFD (/mm^2^)	38.13 (8.23)	33.90 (7.64)	0.011
	CNBD (/mm^2^)	50.07 (14.56)	58.40 (22.70)	0.040
Dendritic Cell	CDC (/mm^2^)	13.55 (8.77)	27.75 (27.55)	0.019

Data are presented as mean (SD) values

CCM, corneal confocal microscopy; ALS, amyotrophic lateral sclerosis; IWL, corneal nerve length in the inferior whorl area; IWDC, dendritic cell density in the inferior whorl area; CNFL, corneal nerve fiber length in the peripheral area; CNFD, corneal nerve fiber density in the peripheral area; CNBD, corneal nerve branch density in the peripheral area; CDC, corneal dendritic cell density in the peripheral area

### Correlations between CCM parameters and disease assessment

**Fig. 2:**
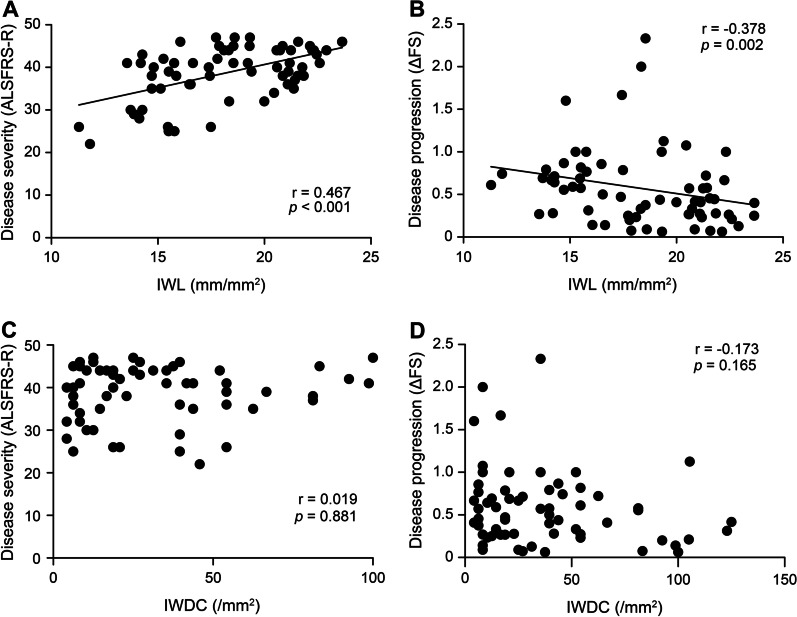
Correlation of CCM parameters in the inferior whorl area with disease severity and progression in ALS. CCM, corneal confocal microscopy; ALS, amyotrophic lateral sclerosis; IWL, inferior whorl length; IWDC, dendritic cell density in the inferior whorl area; disease severity was based on the Revised ALS Functional Rating Scale (ALSFRS-R); disease progression was calculated by ΔFS, the declining rate of ALSFRS-R at assessment (ΔFS = (48-ALSFRS-R)/disease duration from symptom onset to the assessment)

Figure 2 illustrates the correlations between CCM parameters and disease assessments with detailed data shown in Additional file 1: Supplementary Table 2. A significantly positive correlation was found between IWL and disease severity scored by the ALSFRS-R (r = 0.467, *p* < 0.001). Besides, the bulbar sub-scores of ALSFRS-R were also significantly correlated with IWL (r = 0.335, *p* = 0.006). A significantly negative correlation was observed between IWL and disease progression based on ΔFS (r = − 0.378, *p* = 0.002). However, no significant correlation was observed between IWDC and disease severity or progression scores (*p* > 0.05). A relatively weak correlation was found between CNFL and ALSFRS-R (r = 0.282, *p* = 0.022). However, there were no other significant correlations between disease assessments and CCM parameters from the peripheral area, including CNFL, CNFD, CNBD and CDC (*p* > 0.05; Additional file 1: Supplementary Table 2).

### CCM comparisons between patients with and without bulbar involvement

**Fig. 3:**
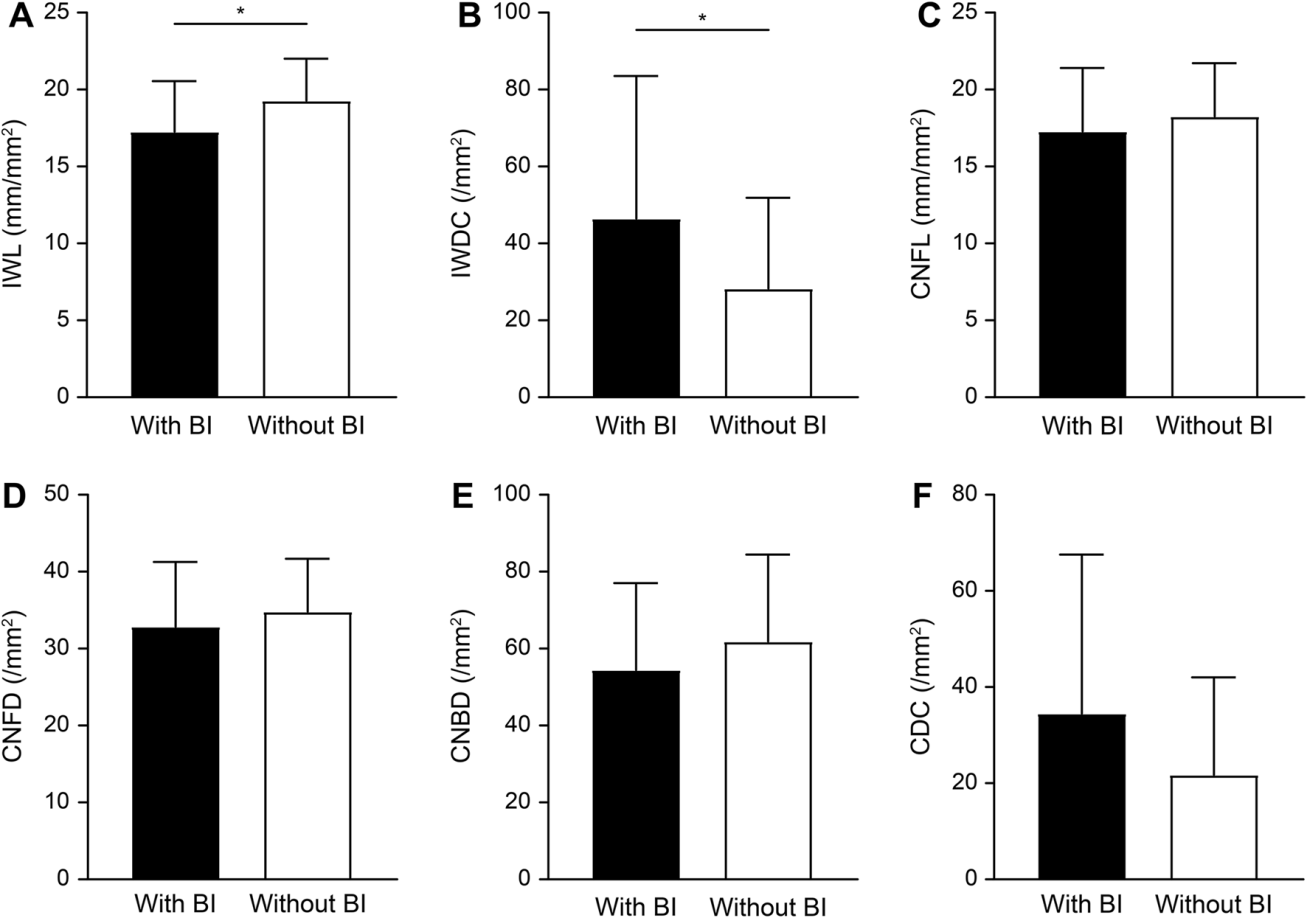
CCM parameters in patients with bulbar involvement and without bulbar involvement of ALS. CCM, corneal confocal microscopy; ALS, amyotrophic lateral sclerosis; IWL, corneal nerve length in the inferior whorl area; IWDC, dendritic cell density in the inferior whorl area; CNFL, corneal nerve fiber length in the peripheral area; CNFD, corneal nerve fiber density in the peripheral area; CNBD, corneal nerve branch density in the peripheral area; CDC, corneal dendritic cell density in the peripheral area; *p < 0.05

When patients were divided into two groups based on bulbar involvement, subgroup analyses showed significant differences in the inferior whorl area but not in the peripheral area (Fig. 3 and Additional file 1: Supplementary Table 3). The patients with bulbar involvement showed significantly lower IWL (*p* = 0.014) and higher IWDC (*p* = 0.043) than the patients without bulbar involvement. Although there were no significant differences in CCM parameters from the peripheral area in the within-case comparisons, the trend of lower nerve fiber length with higher dendritic cell density was similar to the results from the inferior whorl cornea.

## Discussion

This study evaluated the utility of CCM for the quantification of corneal structures in patients with ALS. We compared the typical CCM impairments of patients with healthy controls and further explored its clinical value for the assessment of disease severity in ALS. To our knowledge, this is a pioneering study demonstrating alterations in corneal nerves and dendritic cells in the inferior whorl area of patients with ALS, which could serve as a potential imaging marker for peripheral neuropathy in ALS.

Compared with healthy controls, we identified the lower corneal nerve length and greater corneal nerve complexity in the peripheral area of patients with ALS in our study. This significant corneal nerve loss (decreased CNFL and CNFD) was consistent with the previous studies, which represented the degenerative process in small-fiber nerves with multifactorial pathogenesis including inflammation, metabolism, and abnormal protein deposition [16, 20, 27, 28]. Besides, we found a significantly increased CNBD in patients with ALS, which has also been observed in patients with Parkinson’s disease [29, 30]. It has been proposed that the increased CNBD was a signal of regeneration reflecting a more complicated structure of the nerve terminals [19]. However, this was not identified in other neurological diseases. One interpretation is that the regenerative ability has been decompensated with long disease course, represented by the inherited diseases with cumulative injury since birth (i.e., Friedreich ataxia, and familial amyloid polyneuropathy) [17, 31]. Another interpretation is that the regeneration may less occur in the relatively acute process of the inflammatory diseases (i.e., Behcet’s disease, and multiple sclerosis) [16, 32]. Thus, our CCM results could be explained by the unique dying-back mechanism in ALS, which addresses the compensatory axon sprouting and reinnervation of nerve endings that accompanies degeneration [33]. Additionally, our findings of degeneration and regeneration in corneal nerves are also consistent with previous histopathological studies of small fiber neuropathy in skin biopsy in ALS, which identified similar dual processes in the cutaneous sensory and autonomic nerves [34].

Our study initially demonstrated corneal nerve damage in the inferior whorl area (IWL) of patients with ALS, which had close associations with disease severity assessments in subsequent correlation analyses. IWL has been identified as a sensitive marker for length-dependent impairment in previous studies, especially in diabetic neuropathy [28]. Thus, our positive findings could provide potential evidence indicating a similar impairment pattern of the small fiber nerves in ALS. Physiologically, the corneal nerves follow centripetally migrating growth, which enters the tissue radially and eventually forms a clockwise whorled pattern. Since the typical length-dependent pattern follows impairments along a distal-to-proximal gradient, the inferior whorl area representing the most distal area of the peripheral nerve should show the earliest and most severe damage and correlate with disease progression [14]. Pathological studies using skin biopsy in ALS supported this concept of length-dependent axonopathy, as a more significant reduction in epidermal nerve fiber density was found in the distal calves than in the proximal thighs of patients [10]. Considering the stable location and the marked alterations in the corneal nerve in the inferior whorl area, we suggest that IWL could be a potential imaging biomarker to monitor disease progression and treatment response for ALS.

We also observed increased dendritic cell densities in both the peripheral and inferior whorl regions. However, in patients with direct ocular virus infection, dendritic cell densities were reported to be more than 100 cells/mm^2^ with primary inflammation in the cornea [35]. The magnitude of alterations was much less in our patients with ALS, considering the 10–20 cells/mm^2^ for the secondary immune responses of degeneration. Previous studies have identified inflammation as a consequential response of cell apoptosis in the neurodegenerative cascades of ALS, which has shown great heterogeneity regarding disease progression [36]. Thus, this might explain our unrelated associations between dendritic cell densities and disease severity scores. Further in vitro studies using animal models could provide more insight into the biological features of this concomitant immune response in corneal neuropathy in ALS.

We found that patients with bulbar impairment had more severe corneal impairments in the inferior whorl region than patients without bulbar involvement. Clinically, the presentation of bulbar impairment is found to be especially associated with small fiber neuropathy in several phenotypes of ALS [6, 7], as FOSMN commences with a trigeminal distribution sensory disturbance and progresses by motor weakness spreading along the same craniocaudal distribution with 97% bulbar involvement [8, 9]. The pathological progression pattern also confirmed this result, as a shared pathogenic mechanism between sensory ganglion neurons and motor neurons was confirmed by Sassone et al. [11] in the ALS mouse model SOD1^G93A^. Thus, our positive findings of corneal neuropathy in bulbar-impaired patients provide objective evidence for this association at the patient level. Larger studies are needed to conduct CCM in patients with various phenotypes of ALS to further explore this specific mechanism.

Our study indicated that CCM can be safely performed in ambulatory patients with ALS. In our study, all the patients completed the CCM evaluation with no extra assistance and no adverse events. Considering the latest involved damage of eye movements in ALS, this noninvasive technique is more convenient than the traditional methods to detect small nerve degeneration in ALS. The stable location of the inferior whorl area provides great comparability for the follow-up data with CCM, which might serve as a promising candidate outcome measure for clinical trials. Further studies are needed to confirm its reliability and sensitivity in ALS.

Our study has several limitations. First, the sample size of the participants included in our study was small since it was conducted in a single center. Independent replication of this study with multi-institutional involvement and a larger sample size is essential to support the broader application of CCM in ALS. In addition, dynamic changes in corneal structures should be observed as the disease progresses in a longitudinal study for better efficiency as an imaging biomarker. However, we discovered a close correlation between the inferior whorl parameters and ΔFS at assessment, which could have significant implications for follow-up research. These methods are included in our ongoing work. Besides, clinical evaluation of corneal sensitivity was not included in this study considering the negative results of previous studies in ALS and other neurological diseases [20, 27, 32]. Further research with more elaborate study design is needed to get better understanding of the issue.

In conclusion, we used CCM to quantify nerve fiber loss and dendritic cell increases in both the inferior whorl and peripheral regions of the cornea in ALS. The results complemented the association between corneal small fiber neuropathy and bulbar impairment in ALS. These findings indicated great potential for CCM to serve as a novel, noninvasive, in vivo quantitative tool for peripheral neuropathy detection in ALS.

## Supplementary Information

**Additional file 1**. **Supplementary Table 1.** Baseline characteristics of patients with and without BI in ALS. **Supplementary Table 2.** Correlation of CCM parameters with disease severity and progression in ALS. **Supplementary Table 3.** CCM parameters in patients with and without BI in ALS.

## Data Availability

Data are available for collaborative studies with qualified investigators after inquiry.

## Abbreviations

ALS: Amyotrophic lateral sclerosis
CCM: Corneal confocal microscopy
ALSFRS-R: Revised ALS Functional Rating Scale
IWL: Corneal nerve length in the inferior whorl area
IWDC: Dendritic cell density in the inferior whorl area
CNFL: Corneal nerve fiber length in the peripheral area
CNFD: Corneal nerve fiber density in the peripheral area
CNBD: Corneal nerve branch density in the peripheral area
CDC: Corneal dendritic cell density in the peripheral area
