# Supplementary material for: Small fiber neuropathy for assessment of disease severity in amyotrophic lateral sclerosis: corneal confocal microscopy findings
Source: Orphanet J Rare Dis. 2022 Jan 6;17:7. doi: 10.1186/s13023-021-02157-w (PMC8734326; doi:10.1186/s13023-021-02157-w)
Supplement: Supplementary file 1 — Additional file 1. Supplementary Table 1. Baseline characteristics of patients with and without BI in ALS. Supplementary Table 2. Correlation of CCM parameters with disease severity and progression in ALS. Supplementary Table 3. CCM parameters in patients with and without BI in ALS. [file 13023_2021_2157_MOESM1_ESM.docx]

Supplementary Table 1. Baseline characteristics of patients with and without BI in ALS.

|  | ALS with BI  N = 31 | ALS without BI  N = 35 | *P* value |
| --- | --- | --- | --- |
| Age, y | 49.29 (10.49) | 53.29 (11.60) | 0.149 |
| Sex, no. male/female | 19/12 | 22/13 | 0.896 |
| Disease duration, m | 22.45 (13.04) | 18.43 (11.03) | 0.179 |
| ALSFRS-R | 37.48 (6.83) | 40.07 (5.82) | 0.103 |
| ΔFS | 0.63 (0.52) | 0.52 (0.39) | 0.358 |

ALS: amyotrophic lateral sclerosis; ALSFRS-R: Revised ALS Functional Rating Scale; BI: bulbar involvement; ΔFS: (48-ALSFRS-R)/disease duration from symptom onset to the assessment

Supplementary Table 2. Correlation of CCM parameters with disease severity and progression in ALS.

|  | | Disease severity  ALSFRS-R | | Disease severity (bulbar)  ALSFRS-R bulbar score | | Disease progression  ΔFS | |
| --- | --- | --- | --- | --- | --- | --- | --- |
|  | | R | *P* | R | *P* | R | *P* |
| Inferior whorl area |  |  |  |  |  |  |  |
| Corneal nerve | IWL (mm/mm^2^) | 0.467 | <0.001 | 0.335 | 0.006 | -0.378 | 0.002 |
| Dendritic cell | IWDC (/mm^2^) | 0.019 | 0.881 | -0.179 | 0.150 | -0.173 | 0.165 |
| Peripheral area |  |  |  |  |  |  |  |
| Corneal nerve | CNFL (mm/mm^2^) | 0.282 | 0.022 | 0.095 | 0.449 | -0.153 | 0.221 |
|  | CNFD (/mm^2^) | 0.225 | 0.069 | 0.109 | 0.384 | -0.220 | 0.076 |
|  | CNBD (/mm^2^) | 0.224 | 0.070 | 0.129 | 0.302 | -0.141 | 0.258 |
| Dendritic cell | CDC (/mm^2^) | 0.074 | 0.554 | -0.119 | 0.349 | -0.216 | 0.081 |

ALS: amyotrophic lateral sclerosis; CCM: corneal confocal microscopy; CDC: corneal dendritic cell density in the peripheral area; CNBD: corneal nerve branch density; CNFD: corneal nerve fiber density; CNFL: corneal nerve fiber length in the peripheral area; IWDC: inferior whorl dendritic cell density in the inferior whorl area; IWL: corneal nerve length in the inferior whorl area

Disease severity was based on the Revised ALS Functional Rating Scale (ALSFRS-R); disease progression was calculated by ΔFS, the declining rate of ALSFRS-R at assessment (ΔFS=(48-ALSFRS-R)/disease duration from symptom onset to the assessment)

Supplementary Table 3. CCM parameters in patients with and without BI in ALS.

|  | | ALS with BI  N = 31 | ALS without BI  N = 35 | *P* value |
| --- | --- | --- | --- | --- |
| Inferior whorl area | | | | |
| Corneal nerve | IWL (mm/mm^2^) | 17.26 (3.29) | 19.28 (2.72) | 0.014 |
| Dendritic cell | IWDC (/mm^2^) | 46.49 (37.08) | 28.27 (23.57) | 0.043 |
| Peripheral area | | | | |
| Corneal nerve | CNFL (mm/mm^2^) | 17.29 (4.11) | 18.25 (3.47) | 0.585 |
|  | CNFD (/mm^2^) | 32.86 (8.41) | 34.82 (6.86) | 0.377 |
|  | CNBD (/mm^2^) | 54.44 (22.60) | 61.90 (22.52) | 0.283 |
| Dendritic cell | CDC (/mm^2^) | 34.48 (33.07) | 21.79 (20.22) | 0.126 |

ALS: amyotrophic lateral sclerosis; BI: bulbar involvement; CCM: corneal confocal microscopy; CDC: corneal dendritic cell density in the peripheral area; CNBD: corneal nerve branch density; CNFD: corneal nerve fiber density; CNFL: corneal nerve fiber length in the peripheral area; IWDC: inferior whorl dendritic cell density in the inferior whorl area; IWL: corneal nerve length in the inferior whorl area
